# Agreement Between Reserve Heart Rate, Perceived Exertion and Wint Index During HIIT Using a Low-Cost ANT+ Armband in University Students

**DOI:** 10.3390/s26031049

**Published:** 2026-02-05

**Authors:** Julio Martín-Ruiz, Laura Ruiz-Sanchis

**Affiliations:** 1 Department of Health and Functional Assessment, Catholic University of Valencia, 46900 Valencia, Spain; 2 Department of Sports Management and Physical Activity, Catholic University of Valencia, 46900 Valencia, Spain; laura.ruiz@ucv.es

**Keywords:** Moofit HW401, wearable sensors, health monitoring, HIIT, chronotropic response, psychological demands

## Abstract

High-intensity interval training (HIIT) provides substantial cardiovascular benefits; however, precise monitoring typically requires expensive devices. These systems are feasible in research laboratories but are costly for schools and the fitness industry. Low-cost, validated devices are required to facilitate broader implementation. A cross-sectional study was conducted with 213 students (173 men and 40 women) from the Catholic University of Valencia, Spain. The participants completed an HIIT protocol consisting of five 3 min blocks. Heart rate (HR) was recorded using a Moofit HW401 armband (ANT+ technology). Ratings of perceived exertion (RPE, Omni-Res scale) and the Wint index were also obtained. Pearson correlations were computed between reserve heart rate (HRr), RPE, and Wint index during the warm-up phases (T1, T2) and HIIT, stratified by sex, age, and body mass index (BMI). HRr was strongly correlated with the Wint index (*r* = 0.95, *p* < 0.0001) and moderately correlated with RPE (*r* = 0.235, *p* = 0.001). No significant sex differences were observed (men 83.66 ± 8.18% vs. women 82.31 ± 10.89%; *p* > 0.05). Correlations were weaker in participants with extreme BMI values (n < 10, obese). The Moofit HW401 armband showed consistent agreement between HRr, RPE, and Wint index during HIIT, supporting its practical use for group monitoring in educational settings, pending formal validation against gold standards.

## 1. Introduction

Aerobic physical fitness provides information about the ability to endure moderate- or high-intensity exercise using one or several energy sources, which change depending on the pace of execution [1]. Achieving higher and, most importantly, more sustained intensity levels is an excellent indicator of aerobic fitness [2].

High-intensity interval training (HIIT) is a methodology accessible to all profiles owing to its different variants, which share the maintenance of high intensity and the cardiovascular benefit it provides by reaching levels of aerobic power (95% of VO_2_max) [3,4] or the closest possible value, maximizing the highest acquisition, transport, and utilization of oxygen [5]. In obese adolescents, it can prevent hypertension [6], and in older adults, with appropriate outreach and adherence [7], it improves diastolic function in cases of heart failure, even during short periods of exposure [8], in which a gradual increase in intensity can improve peak VO_2_ [9].

To accurately assess physical condition, isolate the dependent variables in laboratory conditions using devices such as force platforms [10] and accelerometers, which combine kinematics and kinetics [11], obtain the signal with a transducer, and detect gait symmetry or morphological stability [12,13,14], methodological rigor is essential. However, in the recreational field focused on health, this instrumentation is inaccessible because of its cost and participation ratios. In this context, the use of mobile applications to monitor physical fitness indicators has become widespread, as they provide users with immediate information on various variables with minimal instructions, even in a gamified manner, for different purposes [15]. As indicated by Kabir et al. [16], the current reliability of these applications is very low, and the accuracy of the data received is essential for their correct interpretation and subsequent decision-making [17]. It is important that they are intuitive, simple, and allow for quick understanding based on validated instruments to support the planning of conditioning work [18].

In the case of heart rate values, studies have been conducted using devices from well-known brands [19], establishing comparisons between them [20], under laboratory conditions [21,22,23] like ecological [24]. These devices are optical wearables (bracelets, wristbands, etc.) with ANT+ technology, which are widely used today and have made significant findings, such as the correlation between ventilatory thresholds and heart rate [25,26]. It operates in the 2.4 GHz ISM band, with a system of 125 individual channels each with a 1 MHz bandwidth, and a data transmission rate of 4 Hz, optimized for cardiac monitors [27], allowing for the capture of heart rate changes. Transmission is carried out using Gaussian Frequency Shift Keying (GFSK) modulation, emitting frames of 150–190 microseconds to transfer 8 bytes of data, resulting in a throughput of 20 Kbps. The range is 20–30 m under optimal conditions, making it the preferred option for wearable devices [28]. The ANT+ architecture establishes near-zero latencies in the transmission of heart rate data [29], fully meeting the medical requirement of latency under 125 ms, and ensuring that sensors of any brand work with receivers of other brands without additional configuration [30].

Tools such as mobile applications or PC hubs have been designed to monitor heart rate (HR) during group HIIT sessions, providing objective data on internal load (e.g., %HR reserve, Wint index) that surpasses subjective perception (RPE) [31], and are ideal for use in educational and clinical settings [32]. The main problem is that these group monitoring systems are expensive and not easily accessible to the general public [33]. However, its use requires a significant investment in chest straps and annual licensing fees. This limits its widespread adoption, putting it beyond the budgets of some universities and most schools, making it relevant to explore more modest alternatives to enhance accessibility to the technology.

Numerous studies have linked physical activity among university students with variables such as academic performance, according to the number of hours of physical activity [34] or physical fitness based on standard test batteries (Eurofit, Rikli and Jones, or FMS) [35,36]. However, studies have been conducted in which the use of wearables has been widespread for monitoring intensity [37,38,39]. However, a shortage of resources that measure the accuracy of validated, low-cost instruments capable of being integrated into different practice settings has been identified [40]. However, there is insufficient literature on studies that expose university students to high-intensity work, providing the experience, context, and sensations that should be prescribed for high-impact exercise [41].

Therefore, this study aimed to assess the agreement between reserve heart rate (HRr measured by Moofit HW401), ratings of perceived exertion (RPE) and Wint index during a HIIT protocol in university students. Its application could fulfill several objectives, such as determining aerobic fitness levels, extrapolating findings to individuals of the same age outside the degree program, providing students with a reproducible experience for their future careers, and offering an easy-to-implement technological alternative that allows for comprehensive supervision of the physiological response to exercise.

## 2. Materials and Methods

### 2.1. Experimental Approach to the Problem

To assess the suitability of the Moofit HW401 heart rate sensor, a cross-sectional study was conducted using quantitative tests and field evaluations during HIIT development.

Active students from the Faculty of Physical Activity and Sports Sciences participated in the study after providing informed consent. The timing of the test measurements corresponded to the students’ class schedules within the course. The study was designed based on the Declaration of Helsinki to guarantee the fundamental rights of research involving human subjects and was approved by the Ethics Committee of the Catholic University of Valencia (reference number: UCV/2025-2026/002).

### 2.2. Participants

The sample consisted of 213 students enrolled in the bachelor’s degree in physical Activity and Sports Sciences at the Catholic University of Valencia (173 men and 40 women, a proportional ratio in the degree program), Spain, in the subjects Physical Activity and Health and Theory and Practice of Training in Physical Activity. The inclusion criteria were as follows: age between 19–25 years and two subjects, engaging in physical exercise at least four days a week (both in structured sports and activities involving at least 60–90 min daily), and not having performed physical activity on the day of the test or the previous day. The exclusion criteria were as follows: injury at the time of the study, convalescence for less than a month prior to the test, or exemption from practical activities due to a medical prescription.

### 2.3. Procedure

After obtaining informed consent, measurements were performed in two sessions during class periods. In the first session, the main researcher (ISAK-I) measured the participants’ weights and heights. Height was measured using a stadiometer (Seca 213; Hamburg, Germany) with the head positioned in the Frankfurt plane. For weight measurement, a scale (Seca 213, Hamburg, Germany) was used, and the results were recorded after two seconds of stillness.

After this phase, in the second session and before the start, each participant was fitted with a bracelet featuring ANT+ technology (Moofit HW401, Shenzhen, China) with a sampling frequency that was not specified by the manufacturer (approximately 50 Hz). The device was placed on the forearm, and the participant was asked to remain in a relaxed position for at least one minute to record their resting heart rate, captured using specific software (Pulse monitor, Michalowice, Poland), which served as a reference for the expected increases during the main part. Subsequently, a standardized warm-up was conducted, consisting of an initial general preventive section with analytical and isometric exercises to reduce spinal compression and increase muscle stabilization: cat-camel (six repetitions), bird-dog (six repetitions), and front plank (15 s), followed by general joint mobility exercises before the specific warm-up.

### 2.4. Specific Warm-Up

A progressive specific warm-up was conducted, consisting of two parts with an identical structure: a modified Tabata model with three exercise blocks of 20 s each and a 10 s rest. The first part was called T1 (Tabata 1), and the second part was called T2.

**Figure 1:**
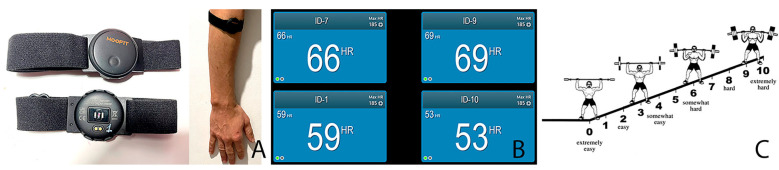
(**A**): Details of the Moofit HW401 sensor and its placement; (**B**): pulse monitor software interface; (**C**): Omni-Res scale.

In T1, the exercises were as follows: (1) pec flys from a prone position, (2) abdominal opening and closing from a prone position, and (3) jump rope. In the case of T2, the exercises included: (1) in-place skipping, (2) floor push-ups, and (3) jumps onto a Swedish gymnastic bench. At the end of each exercise, perceived exertion was displayed using the OMNI-RES scale [42]; each participant was taught the scale and had to indicate the number corresponding to their perception. The evaluator would say the number out loud, and the participant had to confirm it to avoid transcription errors. Heart rate was measured using Pulse Monitor software v.4.2.2. (Figure 1).

Using these data, the reserve heart rate was calculated using the following equation:

$$HRr=Hr\;maximun\;of\;exercise\;*\;\frac{100}{220-age}$$

The Wint aerobic load or index was calculated as follows:

$$IW=Hr\;maximun\;of\;exercise-HR\frac{Basal}{220-age}-HR\;Basal$$

The Wint index is a field calculation used to quantify the internal aerobic load. It helps improve the accuracy of intensity measurements when laboratory resources (such as gas analysis or lactate) are not available; in other words, it serves as a comparative tool. The use of this type of resource is recommended by leading experts [1] and serves as an aid in assessing the approximation of aerobic power during HIIT [43,44,45,46].

### 2.5. Application of HIIT

A HIIT protocol consisting of five blocks of three minutes each was implemented, with a 90 s rest period in between each block. This decision was based on the literature and the principle of accessibility, as the students had different levels of physical fitness. To facilitate the development of work in a university class, traditional cyclical HIIT has been differentiated, and a combined strength workout of an aerobic nature has been implemented to make it accessible to students, this is commonly referred to as HIIRT [47,48]. Therefore, instead of the full seven blocks typical of standard HIIT, a lower number was chosen to ensure high intensity. The recovery interval had a ratio of 1:0.5, as passive recovery was preferred to help participants tackle the remaining blocks with greater motivation. Active recovery (working at the intensity of ventilatory threshold 1) for the same duration as the conditioning phase caused excessive fatigue.

**Figure 2:**
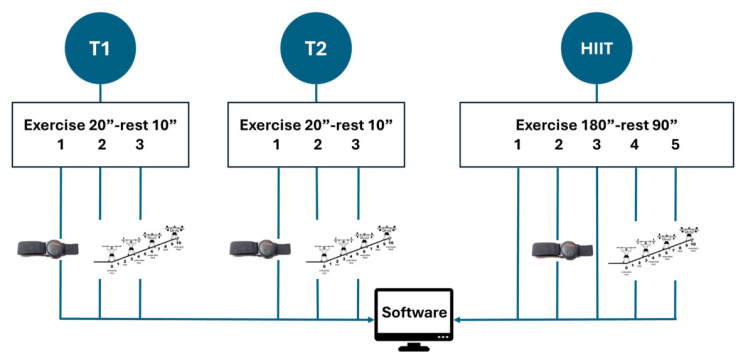
Workflow of warm-up and HIIT workouts.

The exercises used were as follows: (1) squat with partner pass, (2) jump switch on Swedish bench, (3) two steps-jump (landing in a half squat), (4) shadow with ball in pairs, and (5) Jumping Jacks. The decision to use these exercises was based on choosing well-known exercises of a global nature and with highly functional elements, instead of cyclical work, which is more difficult to monitor in standardized class groups, such as in the context of this study. The workflow for warm-up and HIIT is illustrated in Figure 2.

### 2.6. Statistical Analysis

The data were statistically analyzed using descriptive and correlational methods. First, descriptive statistics (mean and standard deviation) were calculated for the anthropometric variables (age, height, weight, body mass index) and physiological variables, including resting heart rate, reserve heart rate, and rate of perceived exertion (RPE) during the two phases of specific warm-up (T1 and T2) and the high-intensity interval training (HIIT) protocol. Additionally, the Wint index calculated for each phase was considered an indicator of the relative load of the effort. The results were stratified by sex.

To compare the anthropometric characteristics and physiological responses between men and women, independent samples Student’s *t*-tests with Welch’s correction were used, since strict equality of variances between groups was not assumed. Sex differences were assessed in the baseline heart rate, reserve heart rate, and perceived exertion during the warm-up and HIIT phases, as well as in the corresponding Wint index values.

To analyze the internal consistency between the objective measurements obtained with the Moofit HW401 armband and the subjective and derived variables, Pearson’s correlation coefficients were calculated between the mean reserve heart rate, perceived exertion, and Wint index in each phase of the protocol (T1, T2, and HIIT). These analyses were performed globally and stratified by sex, age groups (terciles of the distribution), and body mass index categories (normal weight, overweight, and obesity) to explore possible differences in the pattern of associations according to the participants’ profiles. In addition, intraclass correlation coefficients (ICC(3,1) and ICC(3,k)) were calculated for the mean reserve heart rate (%) across T1, T2, and HIIT using a two-way mixed-effects model with a consistency definition to assess the stability of the physiological response across blocks. Along those same lines, Bland–Altman analysis was conducted to assess the agreement and systematic bias between the mean HRR values obtained in T1 and T2.

To estimate the level of cardiorespiratory health from a preventive perspective, the response during HIIT was considered a proxy for the ability to reach intensities close to the maximal aerobic power. In this regard, the mean values of reserve heart rate, perceived exertion, and the Wint index during HIIT were analyzed and compared by sex, age group, and BMI category using Student’s *t*-tests or one-way analysis of variance (ANOVA), as appropriate. Additionally, the criterion for genuine HIIT was defined as the simultaneous fulfillment of a reserve heart rate ≥ 95%, an RPE ≥ 9, and a Wint index ≥ 0.95 [1], to ensure the objectivity of the result when laboratory evidence is lacking. The proportion of participants who met this criterion was compared across sex, age groups, and BMI categories using chi-square test.

Finally, to assess the suitability of the Moofit HW401 armband as a HIIT monitoring tool, the associations between the reserve heart rate during HIIT, perceived exertion, and the Wint index were specifically analyzed by calculating Pearson’s correlations and graphically representing the relationships between these variables using scatter plots with linear regression stratified by sex. Together, these analyses allowed us to determine the extent to which the device consistently reflected the perceived intensity of effort and estimated the relative load during high-intensity interval training.

R software (v. 4.5.2.) was used for all analyses [49] using ggplot2 for visualization [50], with a statistical significance level set at *p* < 0.05 for all tests.

## 3. Results

### 3.1. Descriptive Data of the Sample

First, descriptive statistics were calculated for all anthropometric variables (age, height, weight, and body mass index) and physiological variables (resting heart rate, average reserve heart rate, and subjective perception of exertion during specific warm-up phases 1 and 2 and during the HIIT protocol), stratified by sex. Quantitative variables are expressed as means and standard deviations (Table 1).

To analyze the differences between men and women in the continuous variables, the independent samples Student’s *t*-test (Welch’s correction) was used, since strict equality of variances between groups was not assumed. The level of statistical significance was set at *p* < 0.05. Based on these descriptive and comparative analyses, the associations between the responses measured with the wearable bracelet (resting heart rate) and the subjective and derived variables (perceived effort and Wint index) in the different phases of the protocol were examined.

### 3.2. Correlations Between Reserve Frequency, RPE, and Wint Index by Sex, Age, and BMI

In each phase (Specific Warm-up 1, Specific Warm-up 2, and HIIT), the variables of reserve frequency, perceived exertion, and Wint index were correlated and stratified by sex, age, and body mass index (BMI). The Pearson correlation (*r*) was used in each phase, considering the physiological values (Table 2, Table 3 and Table 4).

In the specific warm-up phase 1 (T1), moderate to high correlations were observed between the average reserve heart rate, subjective perception of effort, and Wint index, especially in men and students with a BMI within the normal weight to overweight range. Overall, a higher reserve heart rate was associated with higher perceived effort scores and higher Wint index values, suggesting a relatively consistent internal response between objective (bracelet) and subjective measures.

In the specific warm-up phase 2 (T2), the pattern of associations was similar, with significant correlations (moderate r) maintained between reserve frequency and RPE and between reserve frequency and the Wint index in most sex and age subgroups. In the extreme BMI categories (e.g., obesity), the correlations tended to be less consistent, probably because of the smaller sample size and greater interindividual heterogeneity in these groups.

During the HIIT protocol, the correlations between mean reserve heart rate, perceived exertion, and the Wint index were generally high and statistically significant in both sexes, indicating that increases in physiological intensity, measured by the wearable armband, were accompanied by parallel increases in perceived exertion and in the relative load estimated by the Wint index. Taken together, these findings demonstrate consistent internal agreement between objective (HRr), subjective (RPE), and derived (Wint) measures of exercise intensity when monitored with the Moofit HW401, supporting its potential as a practical tool for HIIT monitoring in resource-limited educational settings.

### 3.3. Associations Between HRr, RPE, Wint Index and Reliability of HRr Across Blocks

To further examine the stability of the physiological response monitored by the wearable device across the different phases of the protocol, an intraclass correlation analysis was performed on the mean reserve heart rate (%) in T1, T2, and HIIT. The single-measure ICC (ICC(3,1)) indicated moderate consistency between blocks (ICC = 0.55, 95% CI 0.48–0.62, *p* < 0.001), whereas the average-measure ICC (ICC(3,k)) showed good overall consistency when considering the three blocks together (ICC = 0.79, 95% CI 0.73–0.83, *p* < 0.001). Bland–Altman analysis between the T1 and T2 mean HRr values revealed a modest positive bias (6.148), with relatively wide 95% limits of agreement (−15.48% to +27.75%). These results suggest that the pattern of relative HRr responses across the different HIIT-related blocks was reasonably stable at the participant level.

### 3.4. Cardiocirculatory Preventive Value

To approximate the cardiocirculatory preventive value, information from HIIT has been used as a proxy for high aerobic capacity: average reserve heart rate percentage, perceived exertion, and Wint Index in HIIT, bearing in mind that genuine HIIT must simultaneously meet HRr ≥ 95%, RPE ≥ 9, and IW ≥ 0.95. Comparisons of these indicators were made between sexes and age groups using Student’s *t*-test and one-way ANOVA. Students who reached genuine HIIT and those who did not were considered using Student’s *t*-test for continuous variables (HRr, RPE, and IW) and chi-square for proportions (subjects reaching genuine HIIT by age group or BMI category).

Table 5 shows the mean responses during the HIIT protocol by sex. Men and women displayed very similar values in terms of mean reserve heart rate, perceived exertion, and the Wint index, with no statistically significant differences between sexes for any of these variables (Welch’s *t*-tests for independent samples, all *p* > 0.05). This suggests that, in this sample of university students, the relative physiological and perceptual responses to the standardized HIIT protocol were comparable in men and women.

### 3.5. Results by Sex, Age, and Body Mass Index

After applying the chi-squared test to assess the association between sex and genuine HIIT, it was found that there was no significant association between the two variables (*p* = 0.70), just as was the case with age (*p* = 1) and BMI (*p* = 0.95).

Across the entire sample, only a small proportion of participants met the predefined criterion for genuine HIIT (reserve heart rate ≥ 95%, RPE ≥ 9, and Wint index ≥ 0.95). The proportion of responders to genuine HIIT did not differ significantly according to sex, age group, or BMI category (chi-square test, all *p* > 0.05). These findings indicate that reaching a very high relative intensity during the standardized HIIT protocol was uncommon in this cohort and was not clearly explained by sex, age, or body mass index.

No statistically significant differences of great magnitude were observed between men and women in the average reserve heart rate frequency or in the Wint index during HIIT, although in some cases, men tended to reach slightly higher values. Similarly, analyses by age group and BMI category did not show robust patterns indicating a clear disadvantage in terms of the ability to reach intensities close to the maximum aerobic power, aside from small differences that may be attributed to the sample size and individual variability. Overall, the data suggest that, from a preventive perspective, a relevant proportion of university students are capable of reaching high exercise intensities, although not all manage to meet both the most demanding physiological and subjective criteria simultaneously. This indicates that there is room for improvement in cardiorespiratory fitness, which can be addressed through structured, high-intensity training programs.

### 3.6. Suitability of the Moofit HW401 Device According to HIIT Values

**Figure 3:**
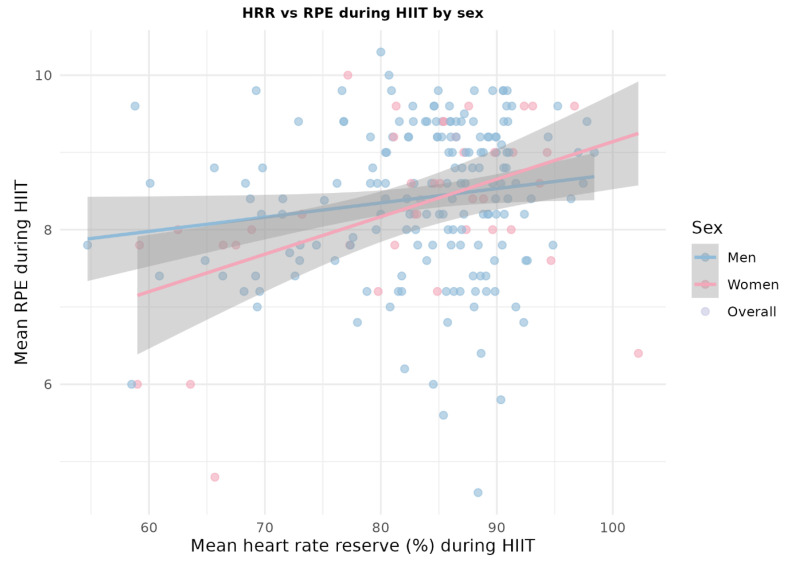
Relationship between mean reserve heart rate (HRr, %) and mean rating of perceived exertion (RPE) during the HIIT protocol, stratified by sex. Each point represents one participant, and the solid lines show sex-specific linear regression fits with 95% confidence intervals. The colors indicate the sex (men and women) and the general trend. The figure illustrates the positive association between the objectively monitored reserve heart rate and perceived exertion during HIIT.

To assess the suitability of the Moofit HW401 armband as a tool for monitoring HIIT, correlations between the Mean HRr with RPE and with the Wint index were considered. Pearson’s correlation between the three HIIT variables was used, stratified by sex, as well as comparisons of mean values of Mean HRr (%) and IW between participants who performed genuine HIIT and those who did not, using Student’s *t*-test (Figure 3).

During HIIT, the correlations between the reserve heart rate measured with the Moofit HW401 armband, subjective perception of effort, and Wint index were generally moderate-to-high and statistically significant. Participants who simultaneously met the criteria for genuine HIIT showed significantly higher reserve heart rates and Wint index values than those who did not reach these thresholds, indicating that the device is sensitive to changes in exercise intensity.

The consistent association between the reserve frequency values recorded by the Moofit HW401 and both subjective indicators (RPE) and derived measures (Wint index) supports the usefulness of the device as a practical tool for monitoring high-intensity interval training in university populations. However, because the device does not directly measure the VO_2_ peak, it should be understood as a complementary internal load monitoring instrument rather than a substitute for laboratory cardiorespiratory testing.

## 4. Discussion

High-intensity work, as applied in the HIIT model, is the standard reference for assessing aerobic fitness in all populations [51], using variables (HRr-RPE) that have been shown to be correlated in various studies [52,53,54]. The paradigm of extensive cyclical work of moderate intensity [55] has limited benefits that mainly contribute to adherence to the practice [56] of physical activity, without the cardiovascular and respiratory benefits that consistent and progressive work provides in interval training [57].

In this study, the Pearson correlations between HRr and IW were high (*r* = 0.95, *p* < 0.0001 in all phases), indicating that the Wint index derived from the HR captured by the Moofit HW401 consistently reflected the relative aerobic load during the practice. Although the correlation between HRr and RPE was more moderate (*r* = 0.23–0.27, *p* < 0.01 in HIIT), perceived exertion is a multidimensional physiological construct that integrates muscle mechanoreceptors, metabolic signals, psychological effort, and environmental information. In contrast, heart rate is a measure of autonomic nervous system activation; previous work in HIIT has documented moderate associations between HR and RPE (*r* = 0.30–0.50) [58,59], suggesting that the observed discordance reflects independent physiological constructs rather than a measurement error of the Moofit HW401 armband.

In the sex-based analysis, correlations were consistent between men and women, with similar *r* values (men HRr-IW *r* = 0.96; women HRr-IW *r* = 0.94, *p* < 0.0001 in both groups). This indicates that the Moofit HW401 optical algorithm was not biased by sex. However, at the extremes of BMI, particularly among participants with obesity (n < 10), the associations tended to be less robust (*r* HRr-IW range 0.85–0.92). This indicates that its performance may be compromised in populations with significant obesity, which is relevant for future clinical applications.

From a clinical perspective, the Moofit HW401 is a useful tool for monitoring relative intensity in recreational contexts, although it has limitations compared with laboratory assessments of relative intensity. The device consistently captured changes in physiological intensity (*r* HRr-IW 0.95) and showed significant correlations with subjective indicators (RPE) and derived measures (Wint). Taken together with the ICC results, the Bland–Altman analysis supports an overall acceptable agreement of mean HRr across blocks, while also highlighting some individual variability in the physiological responses captured by the device. However, because it does not directly measure peak VO_2_, is not calibrated against ventilatory thresholds, and lacks validation against the gold standard, it should be considered a complementary monitoring tool in exercise testing.

The consistent agreement (correlation HRr-IW *r* = 0.95) is comparable to recent studies on optical wearables, such as the Apple Watch Series 6 versus laboratory measurements [60], resulting in similar correlations (*r* ≥ 0.95 at rest, *r* ≥ 0.90 during moderate exercise), with a greater error during intense exercise (*r* ≥ 0.85). Likewise, analysis of the Fitbit Charge 4 in ecological contexts [24] demonstrated high congruence (MAPE ≈ 3–5%) with validated devices. Although the Moofit HW401 has not previously been validated against gold standard devices such as ECG or the Polar H10 chest strap, the internal consistency documented here (*r* = 0.95 HRr-IW) suggests an accuracy comparable to that of other widely adopted commercial devices. Furthermore, it follows some of the INTERLIVE recommendations for validation protocols of PPG wearables [61], such as population heterogeneity or testing context, in this case, a specific HIIT protocol.

Regardless of the findings presented, the research has some limitations, such as the fact that the evaluations are the result of the acute effect, and in future longitudinal studies, it would be possible to assess the stability of the records. The duration of HIIT was deliberately kept shorter, in a pragmatic way, to ensure high intensity during the process; therefore, it should be tested in contexts of 7 to 10 blocks. On the other hand, despite having high concurrent agreement (HRr-IW *r* = 0.95), it would be possible to establish a comparison with other devices of different costs and gold standards to assess the optimal choice for this type of work in different contexts (laboratory, indoor, outdoor, etc.). Along these lines, it would be appropriate to comply with pending premises according to INTERLIVE recommendations, such as using an independently validated criterion measure and indicating standardized error metrics. Therefore, future research should expand on this work with direct comparisons to devices such as the Polar H10 or ECG in samples of 50–100 participants to confirm these results. Finally, these results should be compared with those of samples of different ages and fitness levels to observe whether significant changes occur.

## 5. Conclusions

In the present study, no statistically significant differences were observed between men and women in terms of reserve heart rate or the Wint index, although men had higher values than women.

From a preventive perspective, a significant proportion of university students can achieve high levels of exercise intensity in their daily lives. This reveals the potential for improvement in cardiorespiratory fitness, which can be optimized by designing specific high-impact exercise programs.

Lastly, it can be stated that the increases in physiological intensity, measured with the Moofit HW401 armband, were positively related to perceived exertion and the Wint index, supporting the agreement of the Moofit HW401 device. However, as it does not measure peak VO_2_, it should be used as a complementary and practical monitoring device for tracking high-intensity interval training, pending formal validation against gold-standard tests.

## Figures and Tables

**Table 1:** Descriptive characteristics by sex.

Variable	Men (n = 173)	Women (n = 40)
Age	21.92 ± 3.08	21.93 ± 4.67
Height	1.73 ± 0.07	1.76 ± 0.08
Weight	72.77 ± 9.48	72.83 ± 8.66
BMI	24.28 ± 3.52	23.58 ± 3.40
HR Basal	79.49 ± 14.13	81.70 ± 13.82
Mean HRr (%) T1	68.49 ± 11.50	68.15 ± 15.83
Mean RPE T1	3.91 ± 1.72	3.41 ± 1.60
IW T1	0.47 ± 0.19	0.47 ± 0.23
Mean HRr (%) T2	74.73 ± 10.63	73.92 ± 13.88
Mean RPE T2	5.41 ± 1.68	4.77 ± 1.85
IW T2	0.58 ± 0.18	0.56 ± 0.23
Mean HRr (%) HIIT	83.66 ± 8.18	82.31 ± 10.89
Mean RPE HIIT	8.41 ± 0.99	8.28 ± 1.12
IW HIIT	0.72 ± 0.14	0.71 ± 0.19

Note: HR: Heart rate; HRr: Reserve heart rate; T1/T2: Specific warm-up 1 or 2; RPE: Rating of perceived exertion; IW, Wint Index.

**Table 2:** Pearson correlations between reserve heart rate, perceived exertion and Wint index during warm-up phase 1 (T1).

	Mean HRr (%) T1	Mean RPE T1	IW HR T1
Mean HRr (%) T1	*r* = 1.000 (*p* = −0.0000)	*r* = 0.157 (*p* = 0.0437)	*r* = 0.957 (*p* = 0.0000)
Mean RPE T1	*r* = 0.157 (*p* = 0.0218)	*r* = 1.000 (*p* = −0.0000)	*r* = 0.108 (*p* = 0.1141)
IW HR T1	*r* = 0.957 (*p* = 0.0000)	*r* = 0.108 (*p* = 0.1141)	*r* = 1.000 (*p* = −0.0000)

Note: Values are Pearson’s *r*, with *p*-values in parentheses. The variables were the mean reserve heart rate during T1, mean rating of perceived exertion (RPE) during T1, and Wint index based on the T1 heart rate. Strong positive correlations were observed between the reserve heart rate and the Wint index, and a small but significant association was observed between the reserve heart rate and RPE. HR: Heart rate; HRr: Reserve heart rate; T1: Specific warm-up 1; RPE: Perceived exertion; IW: Wint index.

**Table 3:** Pearson correlations between reserve heart rate, perceived exertion and Wint index during warm-up phase 2 (T2).

	Mean HRr (%) T2	Mean RPE T2	IW HR 2
Mean HRr (%) T1	*r* = 1.000 (*p* = −0.0000)	*r* = 0.272 (*p* = 0.0001)	*r* = 0.947 (*p* = 0.0000)
Mean RPE T1	*r* = 0.272 (*p* = 0.0001)	*r* = 1.000 (*p* = −0.0000)	*r* = 0.235 (*p* = 0.0005)
IW HR T1	*r* = 0.947 (*p* = 0.0000)	*r* = 0.235 (*p* = 0.0005)	*r* = 1.000 (*p* = −0.0000)

Note: Values are Pearson’s r, with *p*-values in parentheses. The variables included the mean reserve heart rate during T2, mean RPE during T2, and Wint index based on the T2 heart rate. There was a strong positive relationship between the reserve heart rate and the Wint index and moderate significant associations between the reserve heart rate and RPE and between the RPE and the Wint index. HR: Heart rate; HRr: Reserve heart rate; T2: Specific warm-up 2; RPE: Rate of perceived exertion; IW: Wint index.

**Table 4:** Pearson correlations between reserve heart rate, perceived exertion and Wint index during the HIIT protocol.

	Mean HRr (%) HIIT	Mean RPE HIIT	IW HR HIIT
Mean HRr (%) HIIT	*r* = 1.000 (*p* = −0.0000)	*r* = 0.235 (*p* = 0.0010)	*r* = 0.951 (*p* = 0.0000)
Mean RPE HIIT	*r* = 0.235 (*p* = 0.0005)	*r* = 1.000 (*p* = −0.0000)	*r* = 0.214 (*p* = 0.0016)
IW HR HIIT	*r* = 0.951 (*p* = 0.0000)	*r* = 0.214 (*p* = 0.0016)	*r* = 1.000 (*p* = −0.0000)

Note: Values are Pearson’s *r*, with *p*-values in parentheses. The variables were the mean reserve heart rate during HIIT, mean RPE during HIIT, and Wint index based on the HIIT heart rate. During HIIT, the reserve heart rate and Wint index showed a strong positive correlation, and both were moderately and significantly associated with perceived exertion. HR: Heart rate; HRr: Reserve heart rate; perceived exertion: IW: Wint Index.

**Table 5:** Mean HIIT responses by sex (mean ± SD) with sex-difference *p*-values.

Variable	Men	Women	*p* Value
Mean HRr (%) HIIT	83.66 ± 8.18	82.31 ± 10.89	0.412
Mean RPE HIIT	8.41 ± 0.99	8.28 ± 1.12	0.549
IW HR HIIT	0.72 ± 0.14	0.71 ± 0.19	0.687

HR: heart rate; HRr: Reserve heart rate; RPE: Rate of perceived exertion; IW: Wint index.

## Data Availability

The datasets used and analyzed during the current study are available from the corresponding author upon reasonable request owing to privacy and ethical restrictions.

## Abbreviations

The following abbreviations are used in this manuscript:

HR	Heart rate
HRr	Reserve heart rate
RPE	Rate of perceived exertion
IW	Wint index
